# Smart NIR-II croconaine dye-peptide for enhanced photo-sonotheranostics of hepatocellular carcinoma

**DOI:** 10.7150/thno.64759

**Published:** 2022-01-01

**Authors:** Shuang Li, Yang Zhang, Xue Liu, Ye Tian, Yi Cheng, Longguang Tang, Huirong Lin

**Affiliations:** 1 State Key Laboratory of Molecular Vaccinology and Molecular Diagnostics & Center for Molecular Imaging and Translational Medicine, School of Public Health, Xiamen University, Xiamen 361102, China.; 2 International Institutes of Medicine, The Fourth Affiliated Hospital of Zhejiang University School of Medicine, Yiwu 322000, Zhejiang, China.

**Keywords:** microenvironment, *in situ* self-assembly, theranostics, NIR-II, HCC

## Abstract

**Background:** Hepatocellular carcinoma (HCC) is associated with high morbidity and mortality rates. The development of novel nanomaterials represents an important direction for precise HCC theranostics. The combination of photothermal and sonodynamic therapy has provided great benefits for HCC therapy. Theranostic agents in the second near-infrared window (NIR-II, 1000-1700 nm) show great prospects because of their extraordinarily high detection sensitivity, resolution, and deep penetration.

**Methods:** A sharp pH-sensitive self-assembling Glypican-3 (GPC3)-binding peptide (GBP) dye, CR-PEG-GBP, was developed as an intelligent nanoprobe for NIR-II imaging and photoacoustic (PA) imaging-guided photothermal therapy (PTT) and sonodynamic therapy (SDT) of HCC.

**Results:** This small molecule assembled nanoprobe exhibited advantageous properties, such as responding to a decrease in pH (from normal tissue (pH 7.4) to the tumor microenvironment (pH ~6.5)) and aggregating - from small nanoprobes (<20 nm at pH 7.4) - into large nanoparticles (>160 nm at pH 6.5 and >510 nm at pH 5.5) that enables enhanced imaging and therapeutic effects. Because CR-PEG-GBP can self-aggregate *in situ* in an acidic tumor microenvironment, it shows high tumor accumulation and long tumor retention time, while being excretable from normal tissues and safe.

**Conclusions:** This intelligent self-assembling small molecule strategy provides a simple yet efficient solution for HCC theranostics and may open up new avenues for designing clinically translatable probes for HCC treatment.

## Introduction

Hepatocellular carcinoma (HCC) is the fourth most common cause of cancer-related deaths worldwide 1, 2. Owing to the high morbidity and mortality of HCC, with an average five-year survival of <15%, advances in strategies for the treatment of this type of cancer have attracted much attention in recent decades 3. In clinical settings, surgical resection is the treatment of choice for the removal of the majority of abnormal lesions in the liver 4. However, accurately removing all liver tumors is a great challenge when using traditional surgical procedures because of the highly infiltrated nature and unclear boundaries of HCC in the liver 5. Recent advances in the development of molecular imaging probes have provided revolutionary solutions, allowing to precisely define the boundaries of liver tumors for surgical navigation. This has resulted in huge clinical benefits in terms of reducing the potential for tumor recurrence and protecting healthy liver tissue and function 6, 7. Specifically, second near-infrared (NIR-II) fluorescence imaging is an ideal intraoperative real-time imaging method because of its low background signal, high detection sensitivity, low price, high safety, and easy operation 8-11. Recently, the combination of the imaging ability and therapeutic potential of photoactive chemical substances, namely sonotheranostics, has drawn much attention in cancer treatment 12. Photoactive therapy serves as a tool for eradiating residual tumor cells that are invisible. Sonodynamic therapy (SDT) is an emerging approach that involves a combination of low-intensity ultrasound and a specialized sonosensitizer that enables the generation of reactive oxygen species (ROS) in a noninvasive manner 13, 14. Unfortunately, the efficiency of SDT is often attenuated by the presence of antioxidants in tumor cells, in which ROS insulating tumor cells are partially neutralized 15. This situation becomes even more difficult in the context of HCC, due to the intrinsically high antioxidative nature of the liver 16. Therefore, advanced sonotheranostics that alter the sensitivity of tumor cells to SDT may greatly improve the therapeutic potential of sonotheranostics.

Tumors are more sensitive to radiotherapy and chemotherapy at 39‒42 °C due to the improved diffusion and penetration of nanoparticles within the tumor and the promotion of oxidation within this temperature range 17, 18. Moreover, combination therapy integrating both photothermal therapy (PTT) and photodynamic therapy (PDT) has had great success in nanomedicine over the decades 19. Therefore, we hypothesized that the sensitivity of tumor cells to SDT could also be altered under conditions of hyperpyrexia. Interestingly, certain small molecular dyes (such as indocyanine green (ICG)), which have both NIR fluorescence and photothermal therapeutic properties, are favorable for use as SDT agents 20. This type of probe can enable the targeted imaging and therapy of tumors through simple modification, giving rise to a theranostic small molecule with clinical transformation potential. However, due to the poor photothermal stability and poor anti-photo-bleaching ability of phthalocyanine dyes, such as ICG, the use of these dyes for long-term intraoperative imaging and treatment is greatly limited 21, 22. Moreover, the rapid metabolism and low uptake of molecular dyes by tumors result in low tumor-to-muscle contrast in imaging studies 23. An alternative solution is to load these molecular dyes into inorganic materials or organic polymers to create complex nanostructures 24. Although nanoparticles are aided by enhanced circulation half-life and resultant improved pharmacokinetics *in vivo*, nanoparticles are easily captured by the mononuclear phagocytic system (MPS) predominantly in the liver, making it difficult to distinguish normal liver tissues from the liver cancer tissue 25. Therefore, an ideal agent for HCC photo-sonotheranostics should meet the criteria of high photo stability, low likelihood of clearance by MPS, and more importantly, sustained retention in liver cancer cells. To this end, we proposed that molecular agents enabling escape from the MPS clearance in the physiological environment but homing in the HCC region by responding to the tumor microenvironment is a promising strategy.

**Figure 1:**
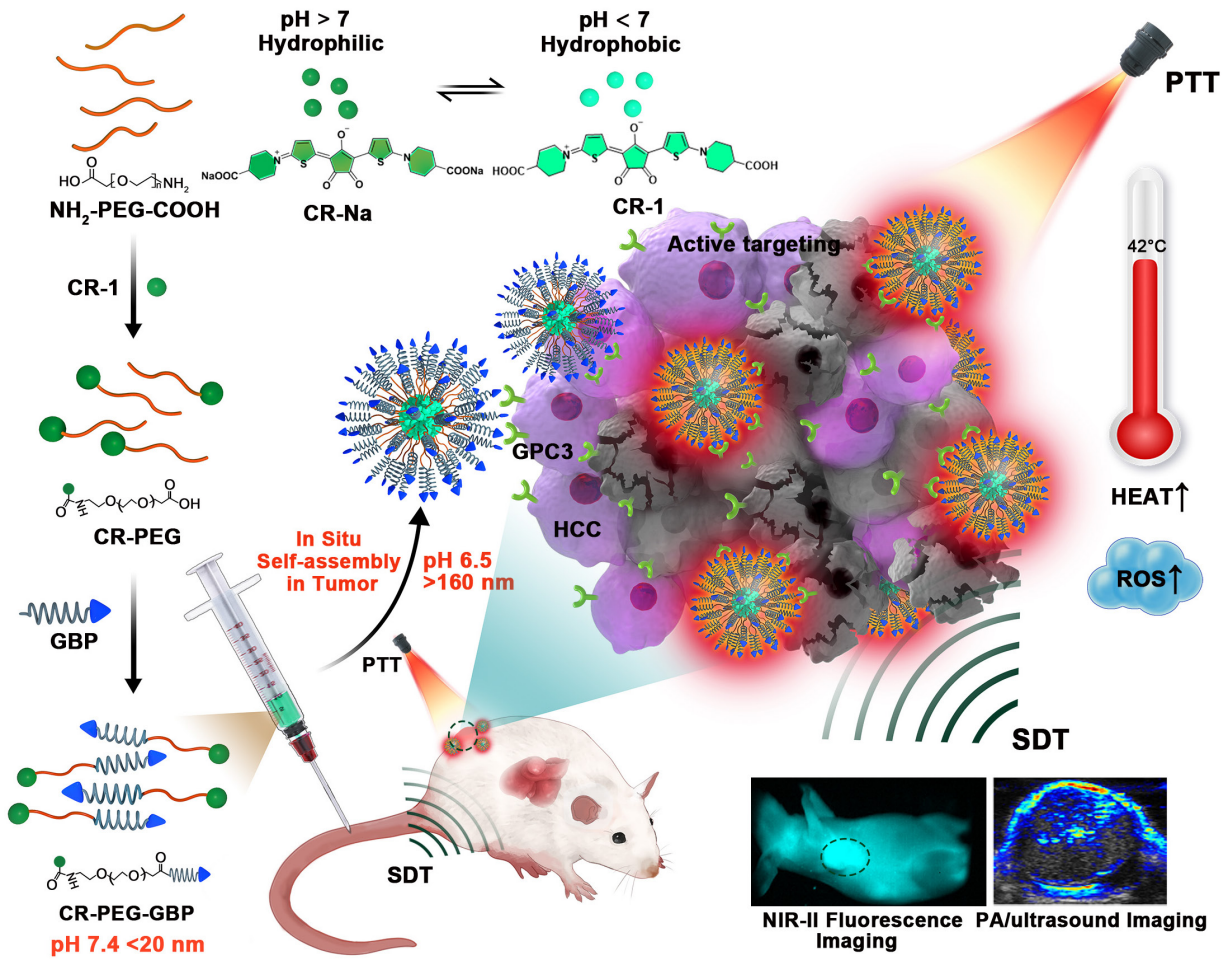
Schematic illustration of the *in situ* self-assembly of CR-PEG-GBP for enhanced HCC photo-sonotheranostics. The croconaine dye CR-1 showed hydrophilic to hydrophobic transformation from neutral (pH > 7) physiological to acidic (pH < 7) condition. In particular, CR-PEG-GBP molecular agents self-assembling into larger nanoparticles in acidic tumor microenvironment exhibit improved tumor retention and enhanced photo-sonotheranostics for HCC, including NIR-II fluorescence and photoacoustic imaging as well as the imaging-guided sonodynamic therapy (SDT) and mild phothermal therapy (PTT).

Herein, we designed a novel croconaine dye (CR-1)-based theranostic agent that exists as monomers or ultra-small nanoparticles (less than 20 nm) under physiological conditions, which can transform into nanoparticles in response to the acidic microenvironment in liver tumors. Self-assembly of CR-1 is mediated by the hydrophilic-to-hydrophobic transformation from weakly alkaline physiological to acidic conditions. More importantly, CR-1 enables both photothermal and sonodynamic synergistic therapy, and can be guided by NIR-II fluorescence imaging and photoacoustic imaging (PAI). We coupled CR-1 with amino-polyethylene glycol_36_-carboxyl (NH_2_-PEG_36_-COOH) and GBP, a polypeptide targeting the GPC3 receptor highly expressed in HCC, to obtain CR-PEG-GBP (**Figure 1**) 26, 27. It is worth noting that HCC treatment greatly benefited from the unique multifunctional properties of CR-PEG-GBP through enhanced photo-sonotheranostics. Because CR-PEG-GBP can self-aggregate *in situ* in an acidic tumor microenvironment, it shows high tumor accumulation and long tumor retention time, while being excretable from normal tissues, and is therefore safe.

## Materials and methods

### General materials

Dulbecco's modified Eagle's medium (DMEM), and fetal bovine serum (FBS) were purchased from HyClone Inc. Ltd. 4-nitrophenyl chloroformate 3-(4,5-9 dimethylthiazol-2-yl)-2,5-diphenyltetrazolium bromide (MTT) and thiophene-2-thiol,4,6-Dia-midino-2-phenylindole (DAPI) were obtained from Sigma Aldrich Co. Ltd. (MO, USA). Reactive oxygen species assay kit (DCFH-DA) were purchased from Sangon Biotech (Shanghai) Co. All other chemicals were purchased from Sigma-Aldrich and used without further purification. Ultrapure water was prepared using a Milli-Q Plus System.

### Synthesis of compound CR-1

The synthetic route of compound CR-1 is shown in Figure S1. Briefly, a mixture of 2.15 g (15 mmol) of methyl isonipecotate, 1.16 g (10 mmol) of thiophene-2-thiol were mixed in toluene (10 mL) and refluxed with vigorous stirring under nitrogen atmosphere for 2 h. After the reaction, the mixture was cooled down to room temperature. The resulting compound methyl 1-(thiophen-2-yl) piperidine-4-carboxylate (1) was purified by column chromatography on silica gel using hexane and ethyl acetate (5/1, v/v) as the eluent. Pale yellow solid was obtained with a yield around 75%. Then, 0.45 g (2 mmol) of product 1 and 10 mL of 0.5 M sodium hydroxide solution were refluxed for 1 h. After cooling to room temperature, the reaction mixture was acidified with 10% acetic acid forming a white precipitate, which was then filtered and dried under vacuum to afford 1-(thiophen-2-yl)piperidine-4-carboxylic acid (2) in the form of a white solid (0.37 g, yield 85%). Next, 211 mg (1 mmol) of compound 2 and 71 mg (0.5 mmol) of croconic acid were dissolved in the mixture of n-butanol and toluene (10 mL, 1:1) and stirred at 120 °C for 1 h. The mixture was filtered, and washed with methanol and dried under vacuum to obtain 230 mg of pure CR780 (yield 90%) as black solid. ^1^H NMR (600 MHz, DMSO-d6): d (ppm) 12.403 (s, 2H), 8.499 (s, 2H), 7.068 (s, 2H), 4.014 (d, *J* = 13.4 Hz, 4H), 3.524 (t, *J* = 11.2 Hz, 4H), 2.670 (s, 2H), 2.041 (d, *J* = 10.2 Hz, 4H), 1.734 (q, *J* = 11.2 Hz, 4H). MS (ESI): m/z = 529.85 [M + H]^+^; calculated m/z = 528.6 for C_25_H_24_N_2_S_2_O_7_.

### Synthesis of CR-PEG-COOH

NH_2_-PEG_36_-COOH (0.536 g, 0.32 mmol), EDC•HCl (0.062 g, 0.32 mmol), and HOBt (0.045 g, 0.32 mmol) were dissolved in 10 mL of CH_2_Cl_2_ and then DIPEA (0.1 mL, 0.6 mmol) was added into the solution. The mixture was stirred in an ice bath for 30 min, followed by the addition of CR-1 (0.16 g, 0.32 mmol). After stirring at room temperature for 24 h, the solvent was evaporated to yield black crude product. The crude was then purified by HPLC to give CR-PEG-COOH (yield 78%). MS (ESI): m/z = 2184.94 [M]^+^; calculated m/z = 2184.

### Synthesis of CR-PEG-GBP

The solution of CR-PEG-COOH (0.218 g, 1mmol) in CH_2_Cl_2_ was added into water and placed in the fume hood overnight to volatile CH_2_Cl_2_, which was self-assembled to CR-PEG-COOH nanoparticles. Then EDC•HCl (0.193 g, 1 mmol) was added into the solution and stirred in an ice bath for 30 min. The GBP peptide (0.119, 1 mmol) and DIPEA (0.2 ml, 1.2 mmol) were added into the above solution and were stirred at room temperature for 24 h. The solvent was evaporated and the solid was purified by HPLC to obtain CR-PEG-GBP (yield 64%). MS (ESI): m/z = 1126.49 [M + NH_4_^+^ + 2H^+^]^3+^/3; Calculated m/z = 3359.

### The pH response self-assembly testing of CR-PEG-GBP

1 mg of CR-PEG-GBP was dissolved in a small amount of acetonitrile, dropped into phosphate buffer salt solution (PBS) with pH values of 7.4, 6.5, and 5.5, respectively. The final concentration of the solution was 1 mg/mL. Dynamic Light Scattering (DLS) and Transmission Electron Microscope (TEM) were used to determine the particle size of the material solution at various pH values.

### Photothermal conversion properties of materials

The photothermal performance of each materials under different power density (1.0 W/cm^2^, 0.7 W/cm^2^, 0.5 W/cm^2^, 0.3 W/cm^2^, 0.1 W/cm^2^) and various concentrations (2.5 μM, 5 μM, 10 μM, 20 μM, 30 μM, and 40 μM) were measured under the laser of 808 nm.

### Sonodynamic properties of materials

Two kinds of ROS detection kits (DCFH-DA and 9,10-dimethylanthracene (DMA)) were used to test the ROS production of materials. The materials were dissolved in PBS solutions placed in a tube and then sonicated with 1MHz, 1.61 W cm^-2^ ultrasound by a multi-function ultrasound device (Selfridge, Beijing), using water as a medium.

### Cytotoxicity Test, Cell uptake, PTT, SDT, and PTT Combined with SDT Effect *in vitro*

They were evaluated *in vitro* using HepG2 cells, Huh7 cells and LO2 cells, which expressed different levels of GPC3 receptors. Cells were purchased from the cell bank of the Chinese Academy of Sciences (Shanghai, China). Cytotoxicity test was studied using the methyl thiazolyl tetrazolium (MTT) assay. Typically, the above different cell lines were seeded into 96-well culture plates at a density of 1 × 10^4^ cells per well. After incubation for 12 h at 37 °C in a CO_2_ incubator, the medium was replaced with fresh medium containing different samples in varied concentrations (0, 10, 20, 40, 60, and 80 µM), and then incubated for another 24 h. The cells were treated with laser irradiation or ultrasound sonication (PTT: 808 nm laser, 0.5 W/cm^2^, 5 min; SDT: 1 MHz, 1.61 W/cm^2^, 5 min). After 24 h, the medium was replaced with culture medium and 10 μL of MTT solution (5 mg/mL, dissolved in PBS) was added to each well. Moreover, the cells were incubated for 4 h. The MTT solution was carefully removed and 150 μL dimethyl sulfoxide (DMSO) was added for 10 min in order to solubilize the violet formazan crystals. The optimal concentration of MTT assay was selected in the cellular uptake test. Cells were treated with different materials in the same concentration (40 μM). 8 h later, diphenyl phenylindole (DAPI) was added to stain the cell nucleus. The cellular uptake was observed by confocal microscope (Olympus, Japan) and the *in situ* self-assembly of different materials in cells was confirmed by TEM. After incubation for 6 h, all of the materials entered the cytoplasm well, cells of every well were treated with the focused ultrasound with a frequency of 1MHz, 30% pulse width and an average intensity of 1.61 W cm^-2^ or treated with 808nm laser (0.5 W/cm^2^) for 5 min separately. For combination therapy, photo laser irradiation was performed at first, then followed with sono-irradiation. Moreover, the cells were incubated for another 18 h. The MTT assay was used to assess cell killing efficiency.

### *In vivo* NIR-II Imaging of CR-1 and CR-PEG-GBP on Hepatocellular Cancer Model

All animal experiments were performed under Animal Ethics Committee of Xiamen University (Fujian, China; acceptance no. XMULAC20120030). BALB/C nude mice were obtained from Beijing Vital River Laboratory Animal Technology Co. Ltd. HepG2 tumor-bearing mice were established by subcutaneously injecting 5×10^6^ cells into the right leg of 6-to-8-week-old athymic nude mice. The HepG2 tumor-bearing mice were anesthetized using isoflurane before placing them on a stage with a venous catheter for injection of imaging agents. For each imaging experiment, at least 3 mice were used per group. All NIR-II images were collected on a two-dimensional InGaAs array (Princeton Instruments). The excitation laser was an 808 nm laser diode at a power density of ~0.15 W/cm^2^. Emission was typically collected with 1000 nm long pass filters and the exposure time is 50 ms. A lens set was used for obtaining tunable magnifications, ranging from 1× (whole body) to 2.5× (high magnification) by changing the relative position of two NIR achromats (200 mm and 75 mm, Thorlabs).

### Photoacoustic/Ultrasound Imaging *In vivo*

When the tumor size reached ∼100 mm^3^, 100 µL of CR-1, CR-PEG, or CR-PEG-GBP (CR-1, 2.5 mg kg^-1^) was intravenously injected into the tumor-bearing mice (n = 5). Time points included one recording before injection (pre) and at 1 h, 2 h, 4 h, 6 h, 9 h, 12 h, and 24 h after injection. The PA signals were performed by Visual Sonic Vevo 2100 LAZR system at a wavelength of 780 nm. The quantified PA intensities were obtained from the region of interests (ROIs).

### *In vivo* PTT, SDT and Their Synergistic Therapy

When the tumor sizes reached ~100 mm^3^, mice bearing HepG2 tumors were intravenously injected with 100 μL of CR-PEG-GBP (2 mM). At 4 h post-injection, the tumors were treated with the 808-nm NIR laser (0.5W/cm^2^, 3 min, diameter of laser spot: 2 mm), ultrasound (1 MHz, 2.68 W/cm^2^, 5 min), or both laser and ultrasound, respectively. To keep the probe cold, cold degassed water (4 °C) was used as the ultrasound couple medium. The temperature of ultrasonic probe showed no significant change (< 3 °C) after treatment. Thermal images of the tumor were taken with a FLIR A×5 camera and quantified by BM_IR software. The mouse body weight and tumor sizes were measured by caliper every other day after treatment and calculated as the volume = AB^2^*0.5, where A was the longer and B was the shorter diameter (mm). The relative tumor volumes were normalized to their initial sizes.

### Statistical Analysis

Experiment results were expressed as mean ± SD. The differences within groups and between groups were determined by two-tailed paired and unpaired Student's t tests, respectively. *P* value <0.05 was considered statistically significant.

## Results and Discussion

### Preparation, characterization, and pH-responsive self-assembly of CR-PEG-GBP

**Figure 2:**
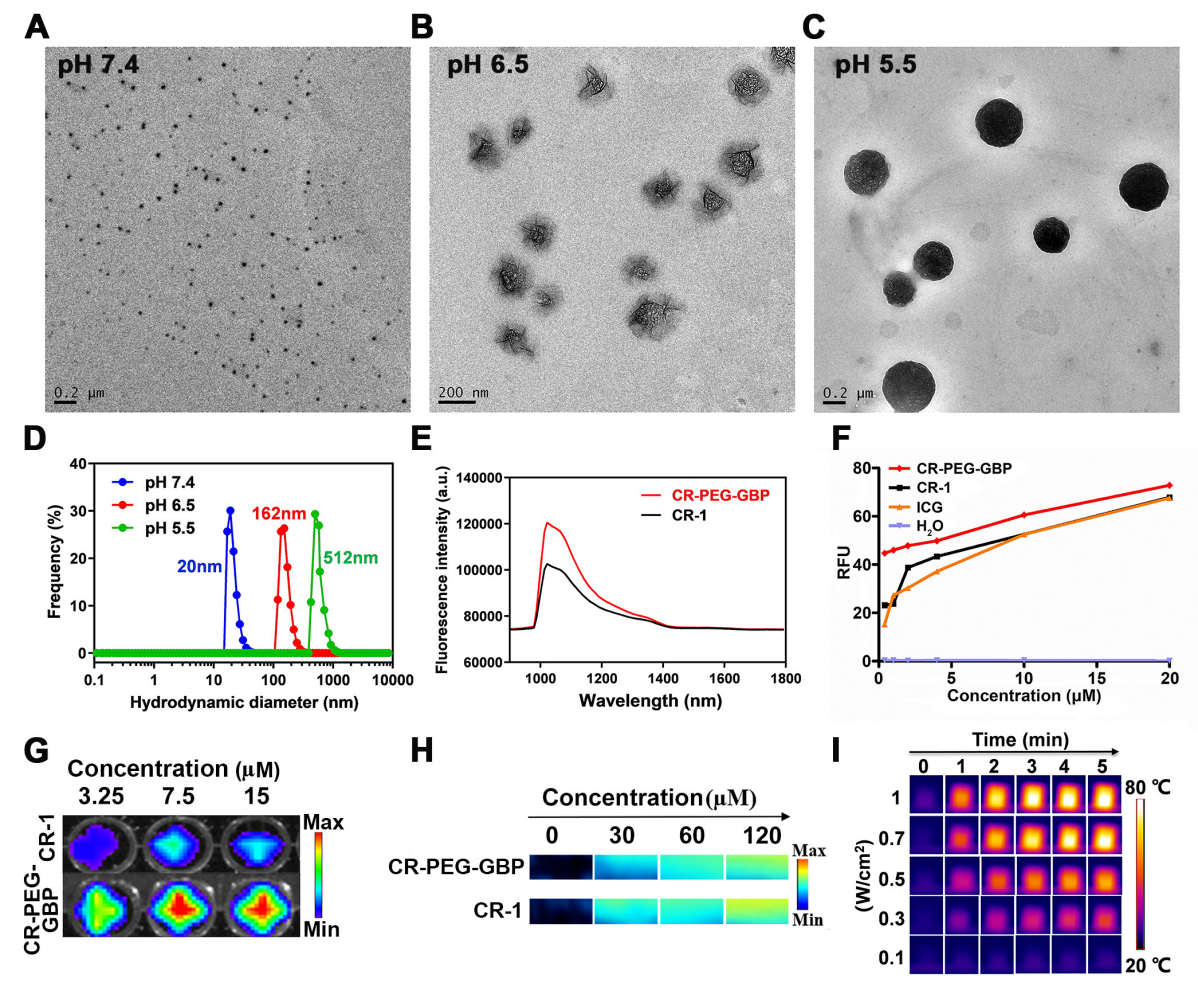
** The evaluation of self-assembly behavior of CR-PEG-GBP under different pH conditions, its optical imaging effect, and its photothermal and sonodynamic transformation efficiency in solution. (A, B, C)** TEM image of CR-PEG-GBP in the solutions with pH values of pH 7.4 (A), 6.5 (B), and 5.5 (C), respectively, prepared in 20 min. **(D)** Corresponding particle diameter of CR-PEG-GBP tested by DLS. **(E)** NIR-II spectrum of CR-1 and CR-PEG-GBP in DMSO (0.2 μM, excitation: 808 nm; 1000 nm LP; 50 ms). **(F)** Sonodynamic effect of CR-PEG-GBP compared with other different materials at different concentrations by testing the fluorescence intensity at 525 nm after ultrasound with DCFH kit (1 MHz, 1.61 W/cm^2^, 5 min). **(G, H)** Fluorescence imaging pictures (G) and photoacoustic imaging pictures (H) of CR-1 and CR-PEG-GBP at different concentrations. (I) Photothermal images of CR-PEG-GBP (40 μM) at different laser (808 nm) energy density and time.

The dye CR-1 was synthesized in three steps and then conjugated with NH_2_-PEG_36_-COOH (MW: 1675) to form CR-PEG-COOH. This was then self-assembled into CR-PEG-COOH NPs with ultra-small nanoparticles. The GPC3 targeting peptide GBP was synthesized according to our previous report, which was then coupled with CR-PEG-COOH NPs to obtain CR-PEG-GBP (**Figure S1**). CR-PEG-GBP is a single molecule, where the molar ratio of CR, PEG, and GBP is 1:1:1. Thus, the percentage of CR-1 in CR-PEG-GBP was 15.7% and that of GBP was 35%. The chemical structures of the above compounds were confirmed by liquid chromatography mass spectrometry (LC-MS) and nuclear magnetic resonance (NMR) (**Figure S2-4**). To simulate the self-assembly behavior of CR-PEG-GBP *in vivo*, we tested the particle size of CR-PEG-GBP under different pH conditions. Transmission electron microscopy (TEM) and dynamic light scattering (DLS) characterizations showed that the particle sizes of CR-PEG-GBP incubated in PBS with different pH values (pH 7.4, 6.5, and 5.5) were 20, 162, and 512 nm, respectively (**Figure 2A-D**). The time-dependent particle aggregation of CR-PEG-GBP in PBS (pH 5.5) was also evaluated by TEM (**Figure S5**). These results demonstrated that CR-PEG-GBP had pH-responsive self-assembly properties, and were expected to self-assemble into large nanoparticles in an acidic environment. The optical properties of CR-PEG-GBP were further evaluated using 808 nm excitation light, which showed an emission wavelength (between 1000‒1400 nm within the NIR-II region) (**Figure 2E**). It is worth noting that the fluorescence intensity of CR-PEG-GBP was stronger than that of CR-1. The quantum yields (QYs) of CR-PEG-GBP and CR-1 in DMSO within the NIR-II region were 0.626% and 0.406%, respectively, i.e., related to carbon nanotubes (QY: 0.03%). Moreover, CR-1 was much more photostable than ICG (**Figure S6**). The absorption peak was between 650‒850 nm, which is within the range of typical PA imaging (**Figure S7a**). The fluorescence intensity and photoacoustic intensity of the samples increased with an increasing concentration within a certain range. Moreover, CR-PEG-GBP showed significantly stronger fluorescence intensity but weaker photoacoustic intensity than CR-1 (**Figure 2G-H, Figure S8**). This phenomenon could be ascribed to the modification of PEG and GBP macromolecular chains, which increased the spatial structure and weakened the aggregation quenching effect of the CR-1 molecules.

To verify whether CR-PEG-GBP has the effect of sonodynamic therapy, we first evaluated ROS production under ultrasonic stimulation with other materials as control samples (H_2_O, CR-1, and ICG) under the same conditions using a 9,10-dimethylanthracene (DMA) kit and a 2',7'-dichlorodihydrofluorescein diacetate (DCFH-DA) kit (**Figure 2F, Figure S7b**). The results showed that CR-1 produced a similar level of ROS, comparable to that of ICG. Surprisingly, CR-PEG-GBP produced significantly larger amounts of ROS than precursor CR-1, which may be due to the fact that modified CR-PEG-GBP lost less heat energy in the excited state molecules. Additionally, the energy conversion efficiency increases according to the principle of energy conservation. Meanwhile, with the increase in concentration, ROS production increased, suggesting that higher drug enrichment will produce more ROS. The mechanism of ROS production in SDT has not been well documented. Several reliable mechanisms are known as sonoluminescence, pyrolysis, ROS production by the collapse of cavitation bubbles, and ROS-independent cytotoxicity. One of the main mechanisms for ROS production for croconaine dye may be ''pyrolysis.'' It has been proposed that during inertial cavitation, the temperature elevation breaks apart the sonosensitizer material to produce radical species (•OH) via water pyrolysis, which reacts with other endogenous substrates to generate ROS. We further tested the photothermal effects of these materials. The heating images and the maximum temperature curve of CR-PEG-GBP (40 μM) under different laser powers (**Figure 2I**) and different illumination times showed that CR-PEG-GBP had excellent photothermal conversion efficiency (**Figure S9 and S10**). As the laser power increased, the temperature increased faster and higher than that of the other control samples, up to 78 °C after irradiation with an 808 nm laser at 1 W/cm^2^ for 5 min. The photothermal stability of CR-PEG-GBP was also investigated and confirmed under NIR irradiation for five cycles (**Figure S11**). To evaluate the pH-responsive behavior of CR-PEG-GBP, we studied the various relevant optical properties under different pH conditions, as shown in **Figure S12**. This shows that most of them change very little, except for ROS generation.

### Specificity, *in situ* self-assembly, and therapeutic effect *in vitro*

**Figure 3:**
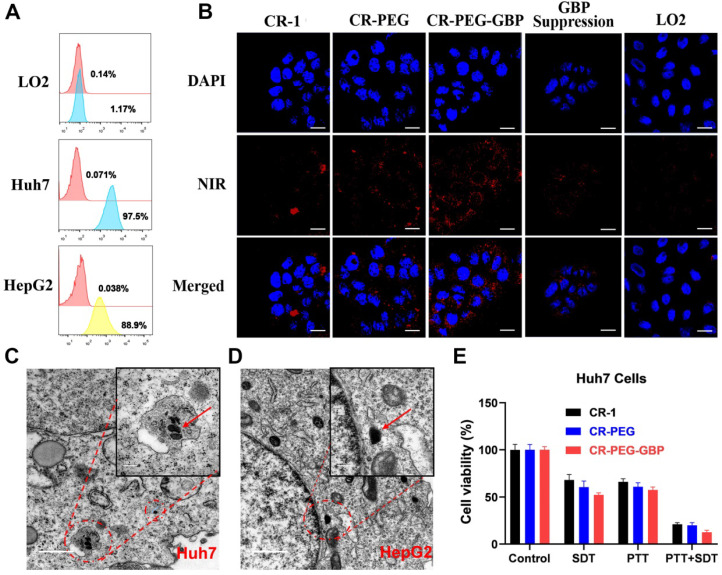
** The evaluation of specific uptake and *in situ* self-assembly behavior of different materials in liver cancer cells and their *in vitro* therapy effects. (A)** Positive rates of GPC3 in different hepatocellular carcinoma cell lines tested by flow cytometry. **(B)** Cellular uptake of different materials in Huh7 cells and that of CR-PEG-GBP in LO2 cells. **(C, D)** TEM images of Huh7 (C) and HepG2 (D) cells treated with CR-PEG-GBP. **(E)** Killing ratio of different materials (40 µM) to Huh7 cell with different therapy methods (PTT: 0.5 W/cm^2^, 5 min; SDT: 1 MHz, 1.61 W/cm^2^, 5 min). Data are mean ± S.D. Two-sided unpaired t-test, n = 4.

Next, we evaluated the specific uptake and *in situ* self-assembly behavior of CR-PEG-GBP in liver cancer cells and its *in vitro* therapeutic effects. Flow cytometry was used to test the binding ability of GPC3 protein-targeted IgG to HepG2, Huh7, and LO2 cells to observe the positive rates of GPC3 protein on the surface of these three cells (**Figure 3A**). The results showed that the expression rate of GPC3 in Huh7 cells (97.5%) was higher than that in HepG2 cells (88.9%). The expression rate in normal liver LO2 cells was only 1.1%. Therefore, the GPC3 receptor can be used as a specific target for hepatocellular carcinoma, and both Huh7 and HepG2 can be used as hepatocellular carcinoma models with high GPC3 expression. Specific cellular uptake was detected by confocal imaging (**Figure 3B, Figure S13**). The results showed that the Huh7 cell uptake of CR-1 was not obvious and uneven, while both CR-PEG and CR-PEG-GBP could enter Huh7 cells. However, after the GPC3 receptor was saturated with peptide GBP, the cell uptake of CR-PEG-GBP was significantly reduced, and there was almost no uptake of CR-PEG-GBP in normal liver LO2 cells. This indicates that CR-PEG-GBP has a good cellular entry effect and can recognize cancer cells with high levels of GPC3, while it is seldom enriched in normal cells. Flow cytometry results (**Figure S14**) showed that materials modified with targeting peptides GBP had higher cell uptake, further proving the GPC3 specific targeting effect of CR-PEG-GBP. CR-PEG-GBP was incubated with HepG2 and Huh7 cells for 8 h. Nanoparticles with a particle size greater than 100 nm were observed in Huh7 and HepG2 cells, and even larger particles were found in lysosomes due to their stronger acidity (**Figure 3C-D**). This indicates that CR-PEG-GBP, a single molecule that we designed, can form self-assembled nanoparticles in cells. ROS production in cancer cells was evaluated after treatment with different compounds and sonicated using ultrasound. The group treated with CR-PEG-GBP showed the highest ROS levels (**Figure S15**). After CR-1, CR-PEG, and CR-PEG-GBP were co-incubated with HepG2 cells and Huh7 cells at different concentrations, the MTT results showed that HepG2 cells (**Figure S16a**) and Huh7 cells (**Figure S16b**) did not show obvious cell death even at the highest concentration (80 μM), indicating little to no cytotoxicity. The cell-killing effect of different treatment methods also showed that the cell killing efficiency of combined therapy (PTT + SDT) was over 80% for both Huh7 cells (**Figure 3E**) and HepG2 cells (**Figure S16c**) after incubation with CR-PEG-GBP (40 μM), which was better than the single SDT (**Figure S16d**) or mild PTT. Therefore, the synergy rate of photosonotherapy was over 80%.

### *In vivo* NIR-II imaging and PA imaging of CR-PEG-GBP on HCC

**Figure 4:**
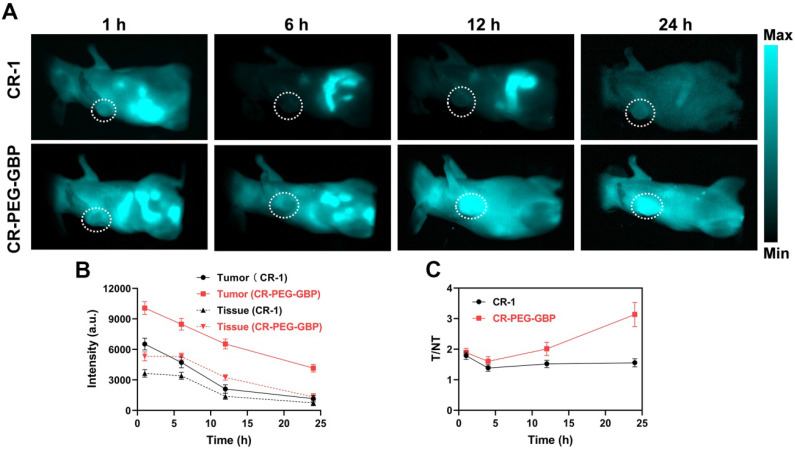
** Evaluation of *in vivo* NIR-II imaging effects of CR-1 and CR-PEG-GBP on hepatocellular carcinoma. (A)** Representative NIR-II imaging results of HepG2 tumor-bearing mice after i.v. injection of CR-1 and CR-PEG-GBP, respectively. **(B)** Quantification of NIR-II imaging results in tumor and normal tissues. **(C)** The ratio of fluorescence signal intensity between tumor (T) and muscle tissue (NT) in different time points (excitation: 808 nm; 1000 nm LP; exposure time: 50 ms). Data are mean ± S.D. (n = 4). T/M = tumor-to-muscle ratio.

NIR-II fluorescence imaging is increasingly widely used in biomedical imaging because of its deeper tissue penetration and higher resolution than NIR-I fluorescence imaging. We found that CR-PEG-GBP showed strong fluorescence intensity within the NIR-II region (900-1400 nm). Therefore, CR-PEG-GBP may be suitable for NIR-II fluorescence imaging to reveal its biological distribution and the optimal time for intratumoral enrichment in order to determine the optimal time range for therapy. As can be seen from the *in vivo* NIR-II fluorescence imaging results, CR-PEG-GBP showed stronger fluorescence intensity than CR-1 at all-time points and showed a significantly higher tumor uptake (**Figure 4**). With the extension of time, stronger fluorescence intensity at tumor sites was observed, and the signal ratio of tumor-to-tissue also increased. However, the signal of CR-1 at the tumor site was diminished. These results indicate that CR-PEG-GBP showed a better tumor-targeting effect and enhanced tumor accumulation and retention than CR-1.

**Figure 5:**
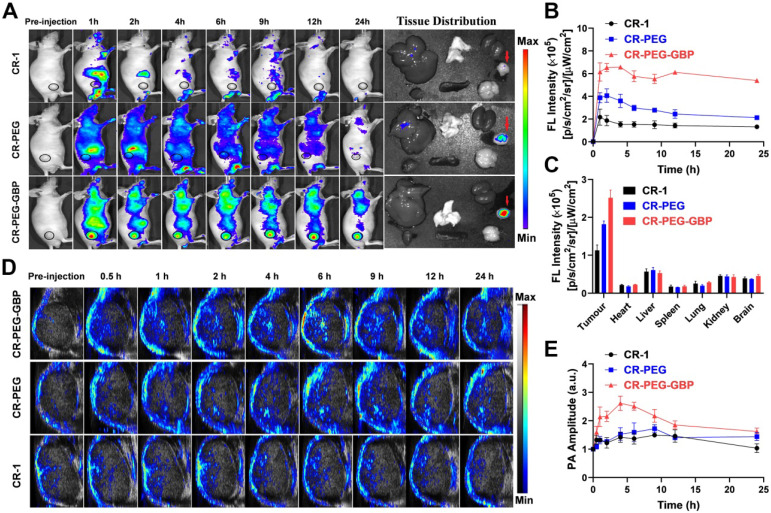
** NIR-I fluorescence imaging and photoacoustic/ultrasound imaging *in vivo*. (A)** Representative systemic fluorescence imaging on HepG2 tumor-bearing mice after the i.v. injection of different compounds and *ex-vivo* tissue fluorescence imaging at 24 h. **(B)** Corresponding mean fluorescence signal intensity at tumor sites. **(C)** The fluorescence signal intensity of tissues at 24 h. **(D)** Representative photoacoustic/ultrasound imaging of tumor *in vivo*. **(E)** Corresponding photoacoustic signal intensity of tumor sites. Data are mean ± S.D. (n = 3).

*In vivo* and *ex vivo* NIR-I fluorescence imaging was also conducted to confirm the biodistribution of CR-PEG-GBP. This result is consistent with that derived from NIR-II imaging (**Figure 5A-B**). The average fluorescence intensity distribution diagram of each tissue (**Figure 5C**) indicated an obvious tumor-targeting effect of CR-PEG-GBP. The fluorescence signal intensity of the tumor was approximately four times that of the normal liver. This indicates that CR-PEG-GBP has potential for use in fluorescence imaging navigation in liver cancer surgery. CR-PEG-GBP also showed stronger photoacoustic signals in tumors than the control groups (**Figure 5D**), indicative of the targeting effect of CR-PEG-GBP. The statistical results showed that after 4 h of administration, the CR-PEG-GBP group showed the highest photoacoustic signal in the tumor (**Figure 5E**). According to the previous experiments, the intensity of the photoacoustic signal was proportional to the concentration of imaging agents; therefore, 4 h after administration was selected as the maximum uptake time of CR-PEG-GBP, at which time the photoacoustic signal was approximately 2.5-fold higher than that of the background.

### *In vivo* PTT and SDT synergistic therapy effects of CR-PEG-GBP

**Figure 6:**
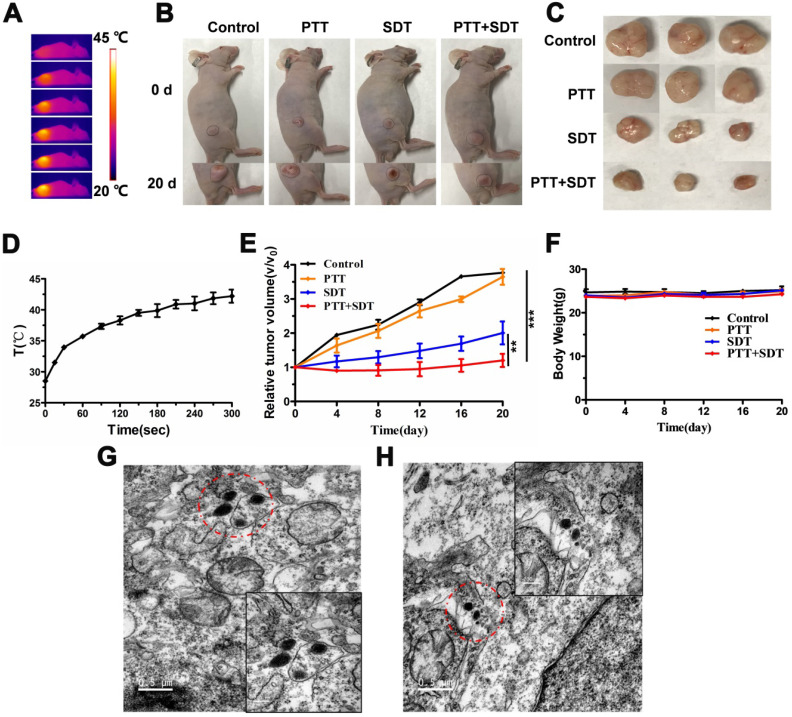
***In vivo* therapy effects. (A)** Thermal images of HepG2 tumor-bearing mice i.v. treated with CR-PEG-GBP (2 mM) and illuminated with 808 nm laser (0.5 W/cm^2^) at 4 h post-injection. **(B)** Representative photos of HepG2 tumor-bearing mice before and after indicated therapy by the i.v. injection of CR-PEG-CBP. **(C)** Images of tumors excised from the above treated mice at 20 days. **(D)** Quantitative analysis of temperature changes in tumor area. **(E)** Tumor growth rate in each group after indicated treatments. Tumor volumes were normalized to their initial size (n = 3 per group). **p < 0.01, ***p < 0.001. **(F)** Body weight changes of mice groups (3 mice for each group) with different treatments (SDT: 1 MHz, 2.68 W/cm^2^, 5 min). **(G, H)** TEM image of CR-PEG-GBP nanoparticles self-assembled in tumor tissue stroma and tumor tissue cells. Data are mean ± S.D. Two-sided unpaired t-test, n = 3 mice.

Based on the *in vitro* results, we evaluated the therapeutic effects of CR-PEG-GBP *in vivo*. From the representative photos of mice and dissected tumors before and after phototherapy, the mouse tumor growth rate after treatment with CR-PEG-GBP (PTT+SDT) was significantly lower than that of the control group (**Figure 6A-C**). Compared with other groups, the tumor growth rate of the PTT+SDT group was significantly inhibited within 20 days, which further proved that CR-PEG-GBP could better inhibit tumor growth *in vivo* with mild PTT-enhanced SDT (**Figure 6D-E**), as well as prolong survival time in tumor-bearing mice (**Figure S17**). The body weight of the mice treated with CR-PEG-GBP showed little change within 20 days (**Figure 6F**), and there was no obvious abnormality in the biosafety evaluation by H&E staining (**Figure S18**), indicating that CR-PEG-GBP had no obvious toxicity in the mouse model. The TEM images of tumor tissue sections dissected at 24 h after CR-PEG-GBP injection showed that CR-PEG-GBP could assemble into nanoparticles with a particle size greater than 100 nm in the tumor stroma and within tumor cells of the tumor tissue (**Figure 6G-H**). These results verified that CR-PEG-GBP could form assembled nanoparticles in response to the tumor microenvironment and was promising for targeted imaging and therapy of HCC.

## Conclusion

In this study, we designed a novel croconaine-based GPC-3 targeting agent, CR-PEG-GBP, to achieve NIR-II fluorescence and PA imaging-guided PTT and SDT for HCC photo-sonotheranostics. Upon actively targeted homing in HCC, the CR-PEG-GBP molecules underwent *in situ* self-assembly into large-sized nanoparticles because of the hydrophilic-hydrophobic transformation triggered by the acidic tumor microenvironment. This phenomenon led to greatly improved tumor retention and enhanced photo-sonotheranostics for HCC treatment. As a result, it showed favorable PTT and SDT synergistic therapeutic effects for HCC guided by NIR-II fluorescence and PA imaging. This study provides a simple yet efficient solution for HCC theranostics. More importantly, the results presented in this study demonstrate that the simple synthetic procedure of the CR-PEG-GBP probe holds great potential for clinical translation.

## Supplementary Material

Supplementary methods and figures.
