# Supplementary material for: Smart NIR-II croconaine dye-peptide for enhanced photo-sonotheranostics of hepatocellular carcinoma
Source: Theranostics. 2022 Jan 1;12(1):76–86. doi: 10.7150/thno.64759 (PMC8690925; doi:10.7150/thno.64759)
Supplement: Supplementary file 1 — Supplementary methods and figures. [file thnov12p0076s1.pdf]

Supporting Information

Smart NIR-II croconaine dye-peptide for enhanced photo-sonotheranostics of hepatocellular carcinoma

Shuang Li[1], Yang Zhang[1], Xue Liu[1], Ye Tian[1], Yi Cheng[1], Longguang Tang[2,✉], Huirong Lin[1,✉]

1. State Key Laboratory of Molecular Vaccinology and Molecular Diagnostics & Center for Molecular Imaging and Translational Medicine, School of Public Health, Xiamen University, Xiamen 361102, China

2. International Institutes of Medicine, The Fourth Affiliated Hospital, Zhejiang University School of Medicine, Yiwu 322000, Zhejiang, China.

✉Corresponding authors
E-mail addresses: tanglongguang@zju.edu.cn (L. Tang); HuirongLin@outlook.com (H. Lin)

Synthesis of compound CR-1. The synthetic route of compound CR-1 is shown in Figure S1. Briefly, a mixture of 2.15 g (15 mmol) of methyl isonipecotate, 1.16 g (10 mmol) of thiophene-2-thiol were mixed in toluene (10 mL) and refluxed with vigorous stirring under nitrogen atmosphere for 2 h. After the reaction, the mixture was cooled down to room temperature. The resulting compound methyl 1-(thiophen-2-yl) piperidine-4-carboxylate (1) was purified by column chromatography on silica gel using hexane and ethyl acetate (5/1, v/v) as the eluent. Pale yellow solid was obtained with a yield around 75%. Then, 0.45 g (2 mmol) of product 1 and 10 mL of 0.5 M sodium hydroxide solution were refluxed for 1 h. After cooling to room temperature, the reaction mixture was acidified with 10% acetic acid

forming a white precipitate, which was then filtered and dried under vacuum to afford 1-(thiophen-2-yl)piperidine-4-carboxylic acid (2) in the form of a white solid (0.37 g, yield 85%). Next, 211 mg (1 mmol) of compound 2 and 71 mg (0.5 mmol) of croconic acid were dissolved in the mixture of n-butanol and toluene (10 mL, 1:1) and stirred at 120 ℃ for 1 h. The mixture was filtered, and washed with methanol and dried under vacuum to obtain 230 mg of pure CR-1 (yield 90%) as black solid. 1H NMR (600 MHz, DMSO-d6): d (ppm) 12.403 (s, 2H), 8.499 (s, 2H), 7.068 (s, 2H), 4.014 (d, J = 13.4 Hz, 4H), 3.524 (t, J = 11.2 Hz, 4H), 2.670 (s, 2H), 2.041 (d, J = 10.2 Hz, 4H), 1.734 (q, J = 11.2 Hz, 4H). MS (ESI): m/z = 529.85 $[M + H]^+$; calculated m/z = 528.6 for $C_{25}H_{24}N_2S_2O_7$.

Synthesis of CR-PEG-COOH. NH_2-PEG_{36}-COOH (0.536 g, 0.32 mmol), EDC•HCl (0.062 g, 0.32 mmol), and HOBt (0.045 g, 0.32 mmol) were dissolved in 10 mL of CH_2Cl_2 and then DIPEA (0.1 mL, 0.6 mmol) was added into the solution. The mixture was stirred in an ice bath for 30 min, followed by the addition of CR-1 (0.16 g, 0.32 mmol). After stirring at room temperature for 24 h, the solvent was evaporated to yield black crude product. The crude was then purified by HPLC to give CR-PEG-COOH (yield 78%). MS (ESI): m/z = 2184.94 $[M]^+$; calculated m/z = 2184.

Synthesis of CR-PEG-GBP. The solution of CR-PEG-COOH (0.218 g, 1mmol) in CH_2Cl_2 was added into water and placed in the fume hood overnight to volatile CH_2Cl_2, which was self-assembled to CR-PEG-COOH nanoparticles. Then EDC•HCl (0.193 g, 1 mmol) was added into the solution and stirred in an ice bath for 30 min. The GBP peptide (0.119, 1 mmol) and DIPEA (0.2 ml, 1.2 mmol) were added into the

above solution and were stirred at room temperature for 24 h. The solvent was evaporated and the solid was purified by HPLC to obtain CR-PEG-GBP (yield 64%). MS (ESI): m/z = 1126.49 [M + NH_4^+ + $2H^+$]$^{3+}$/3; Calculated m/z = 3359.

Supplementary figures

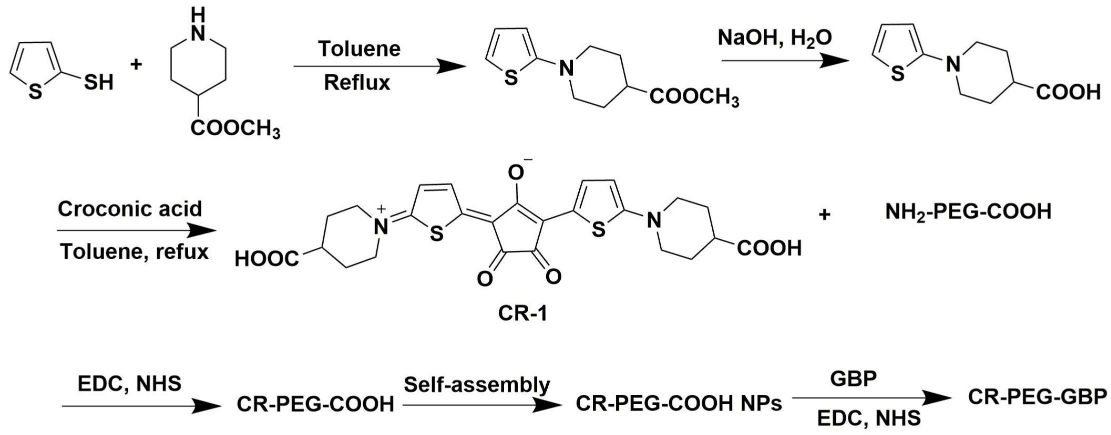

Figure S1. The synthetic routes of CR-PEG-GBP.

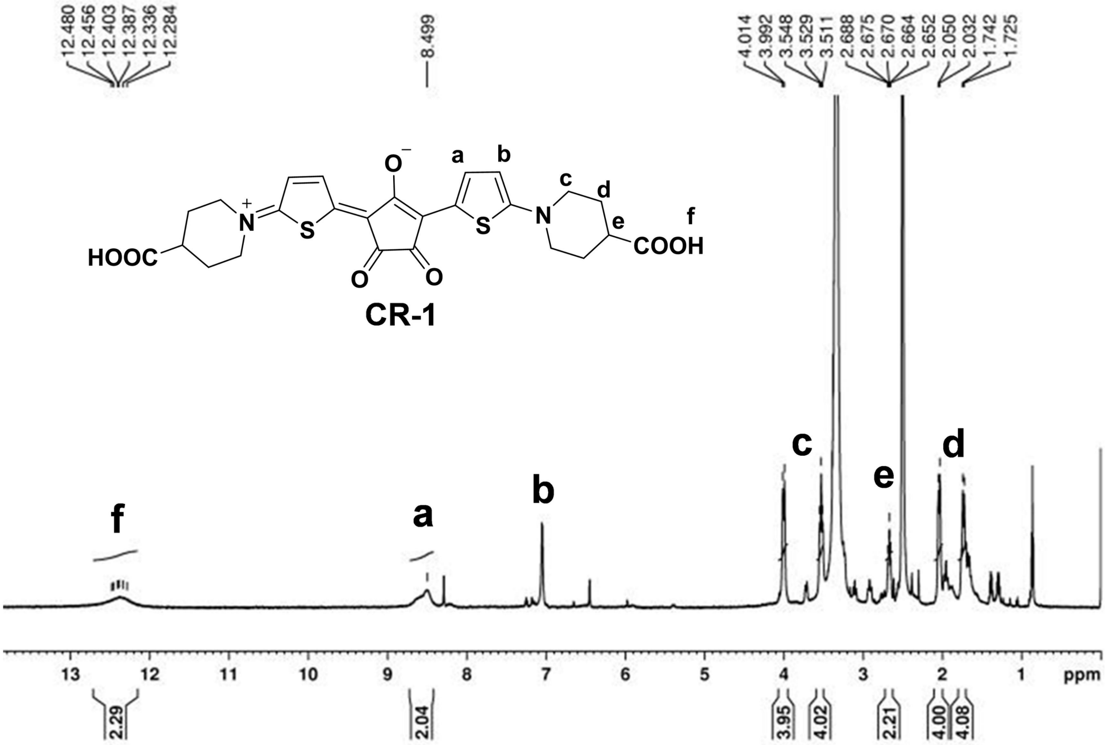

Figure S2. ^{1}H NMR spectrum (in d_6-DMSO) of compound CR-1.

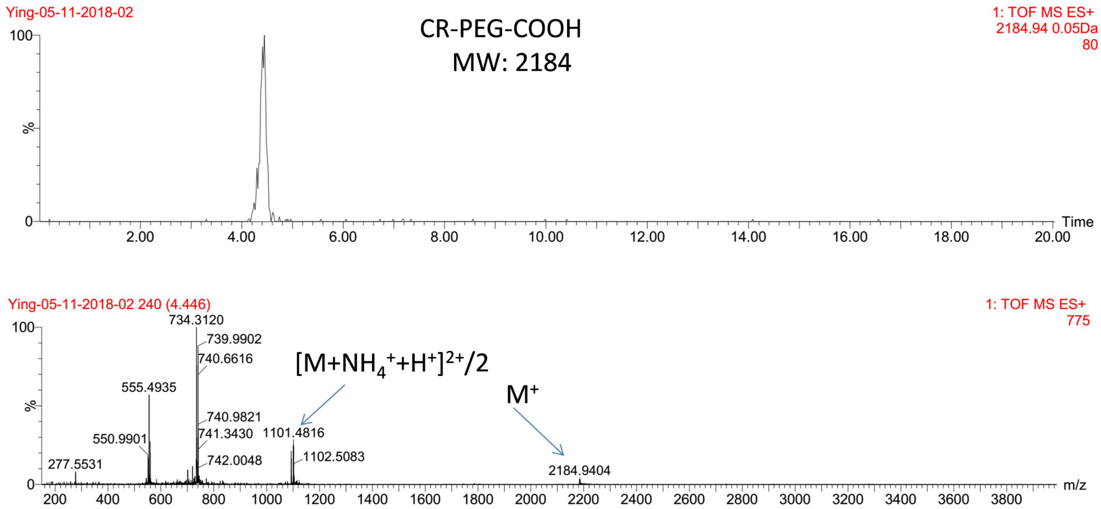

Figure S3. LC-MS spectrum of compound CR-PEG-COOH.

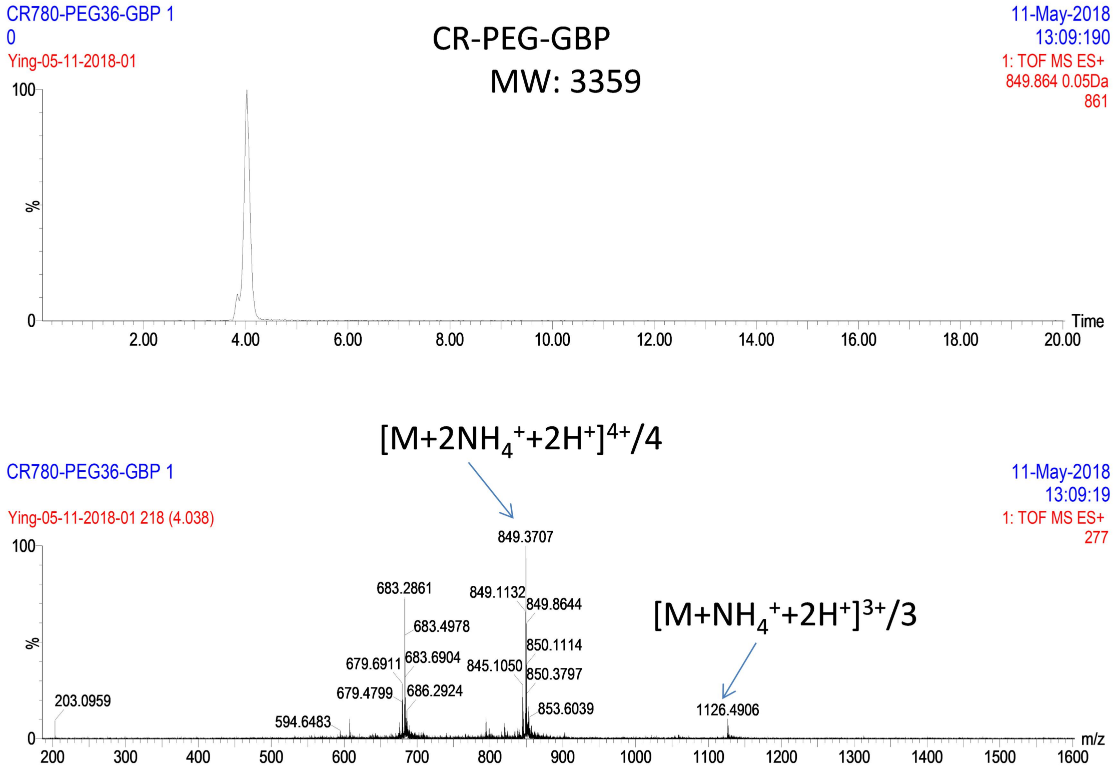

Figure S4. LC-MS spectrum of compound CR-PEG-GBP.

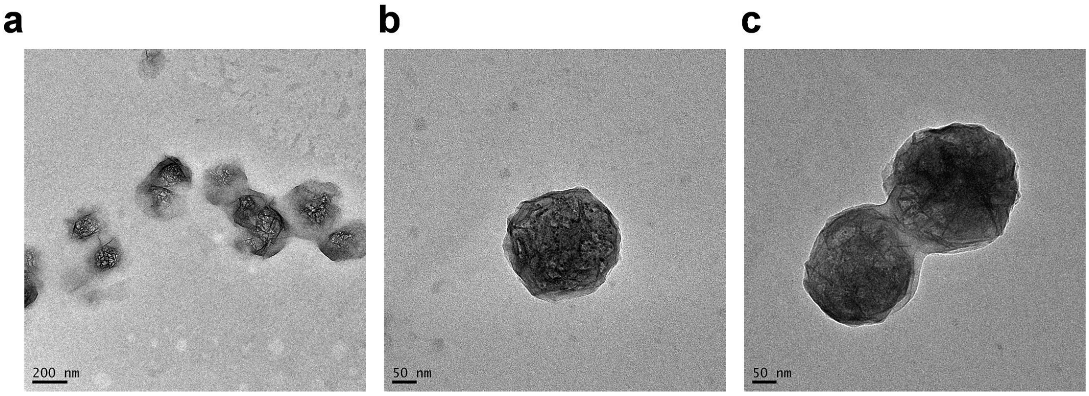

Figure S5. Time-dependent particle aggregation of CR-PEG-GBP incubated in the solution with pH value of 5.5 for 5 min (a), 10 min (b), and 20 min (c), respectively.

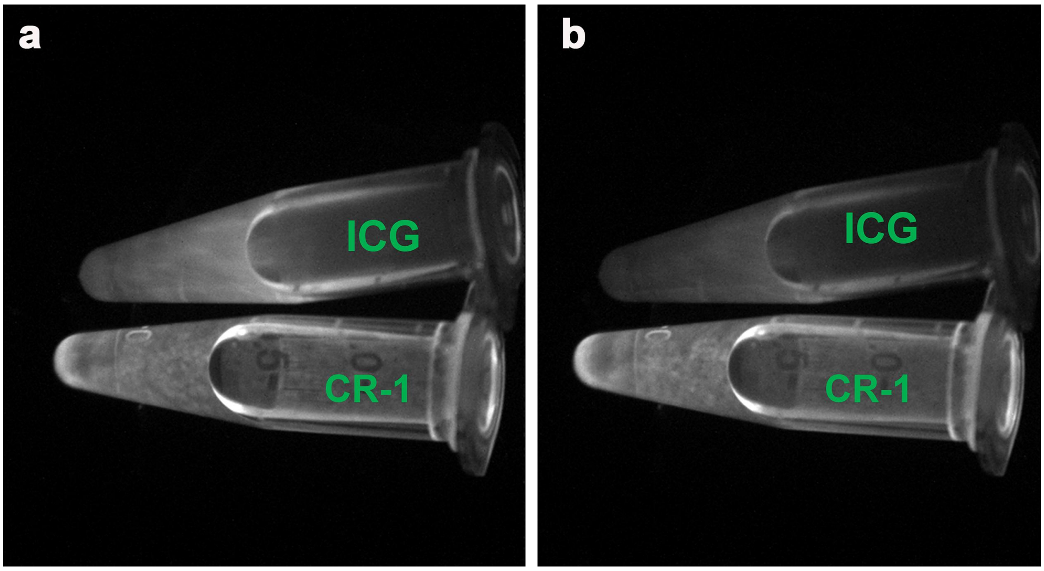

Figure S6. Photo-stability test of CR-1 and ICG in DMSO treated with 808 nm laser. NIR imaging was conducted (a) at the beginning and (b) 30 min after the laser treatment.

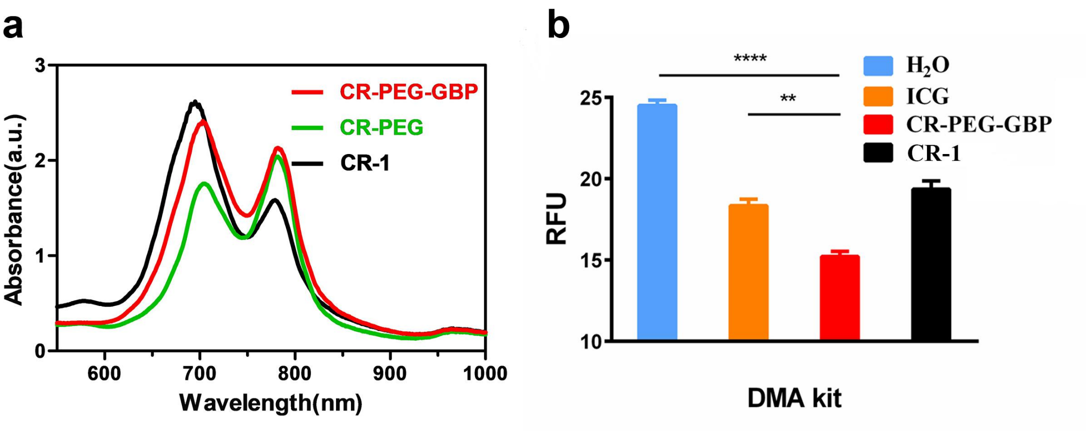

Figure S7. (a)Vis-NIR spectra of indicated compounds in PBS. (b) Sonodynamic

effect evaluation by testing the fluorescence intensity of 20 μM different materials at 434 nm after ultrasound with DMA kit (SDT: 1 MHz, 1.61 W/cm^2, 5 min). **P < 0.01, ***P < 0.001

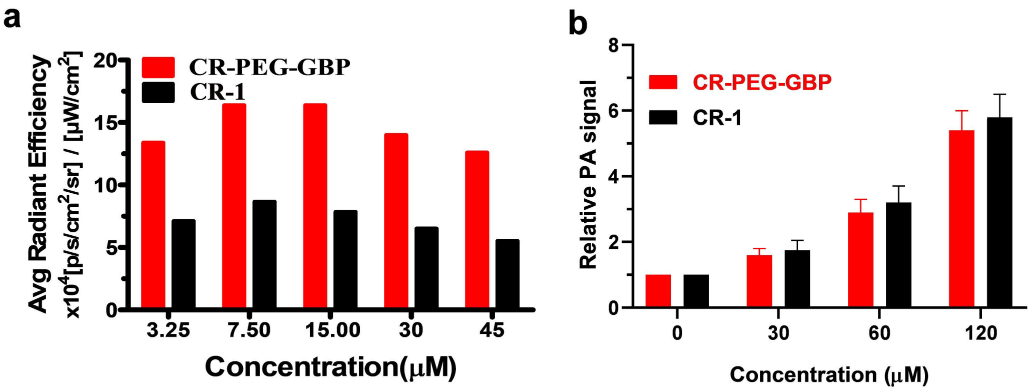

Figure S8. (a) Quantification of corresponding fluorescence intensities of CR-PEG-GBP and CR-1 at different concentrations in PBS. (b) Photoacoustic signal strength of materials at different concentrations in PBS.

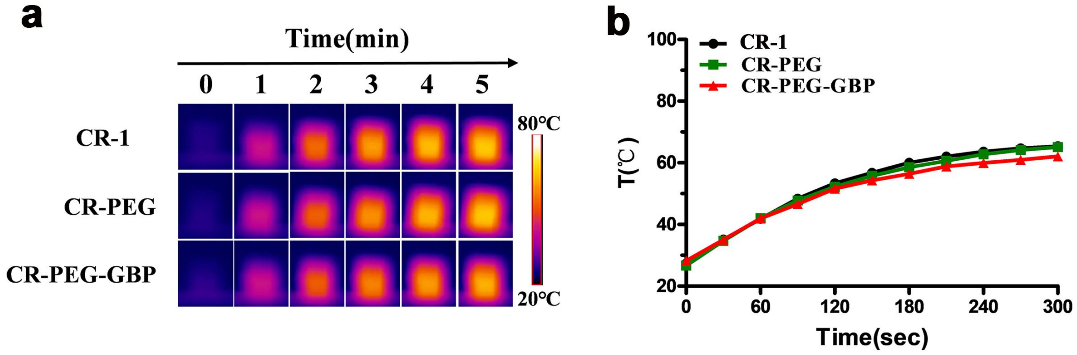

Figure S9. (a) Thermal images of indicated samples (40 μM) illuminated with 808 nm laser (0.5 W/cm^2). (b) Corresponding quantitative analysis of temperature changes of different samples.

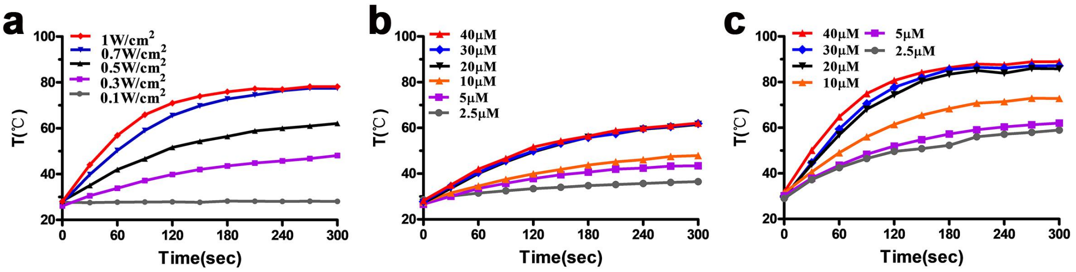

Figure S10. (a) Maximum temperature change curves of 40 μM CR-PEG-GBP at different power and time. (b) and (c) Maximum temperature change curves of CR-PEG-GBP in different concentrations treated with 808 nm laser at 0.5 W/cm^2 (b)

and 1 W/cm^2 (c).

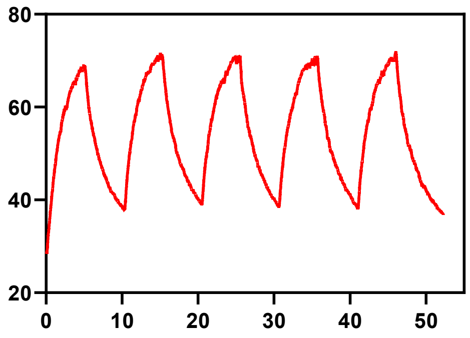

Figure S11. Photothermal stability test of CR-PEG-GBP under NIR irradiation for five cycles.

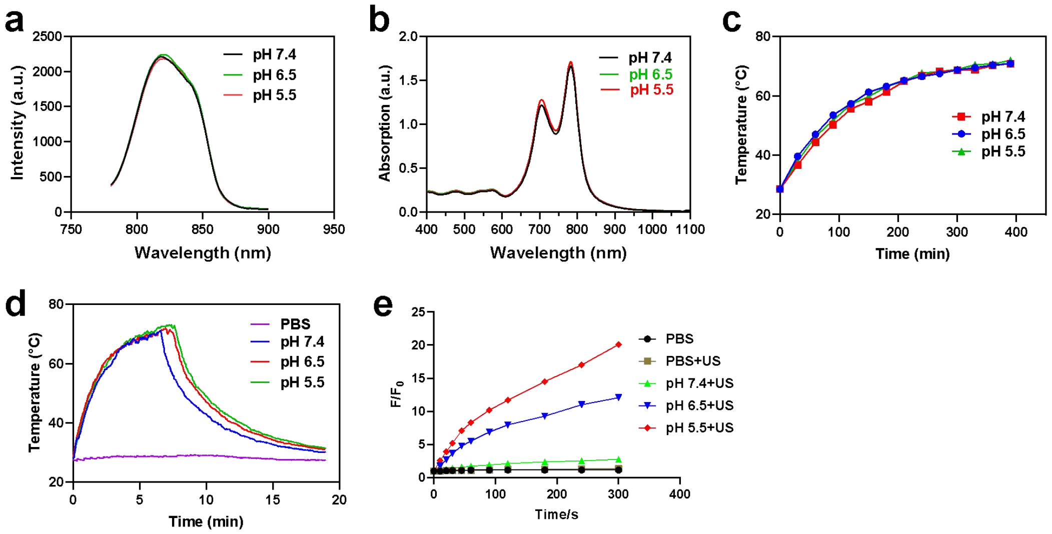

Figure S12. The optical properties of CR-PEG-GP under different pH conditions. It shows that the fluorescence (a), absorbance (b), and photothermal properties (c and d) of CR-PEG-GBP do not change much in different pH conditions, while the ROS production of CR-PEG-GBP treated with ultrasound is increased under the acidic condition (e).

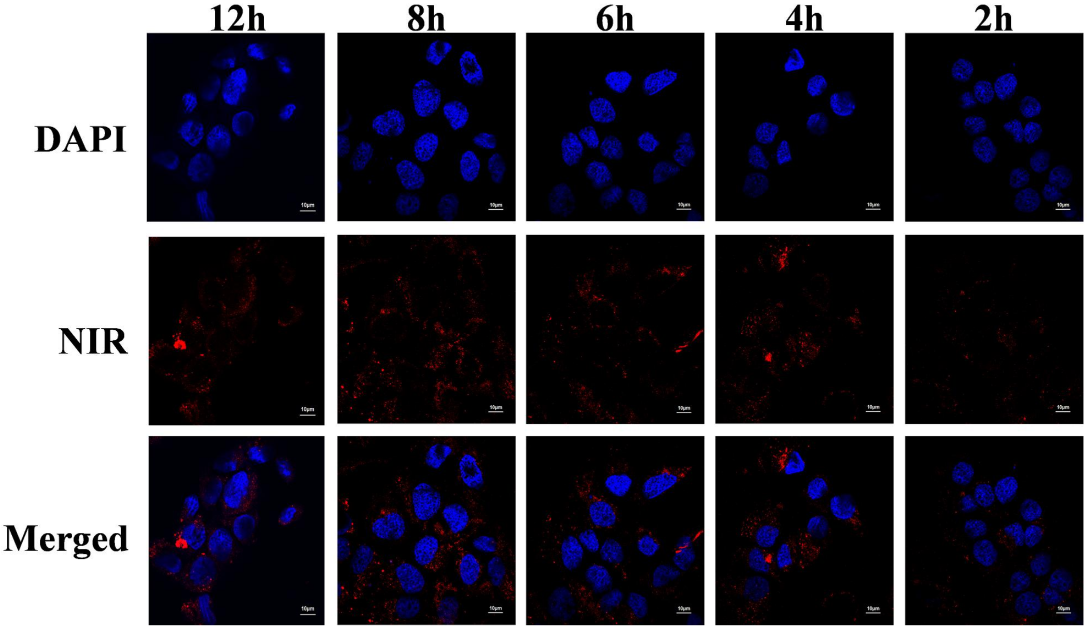

Figure S13. Huh7 Cellular uptake of CR-PEG-GBP (15 μM) in different time points tested by confocal microscopy.

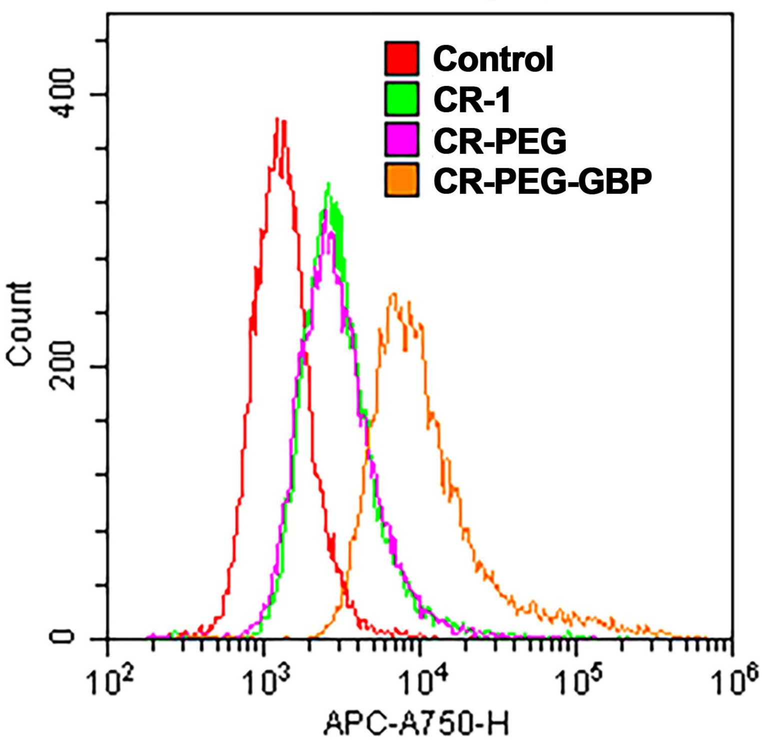

Figure S14. The Huh7 cell uptake of different materials monitored by flow cytometry.

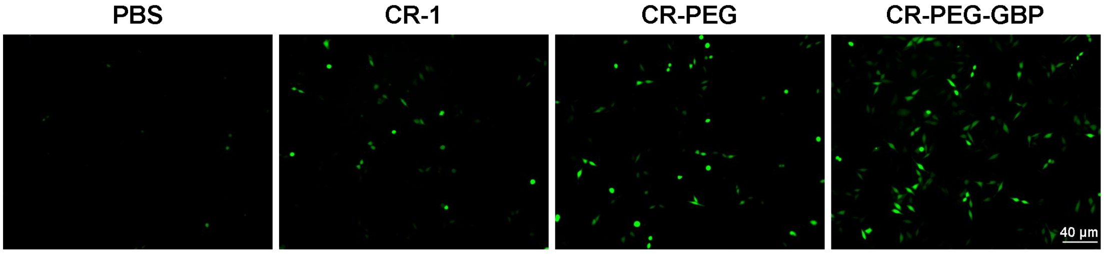

Figure 15. The ROS production in cancer cells after different treatments.

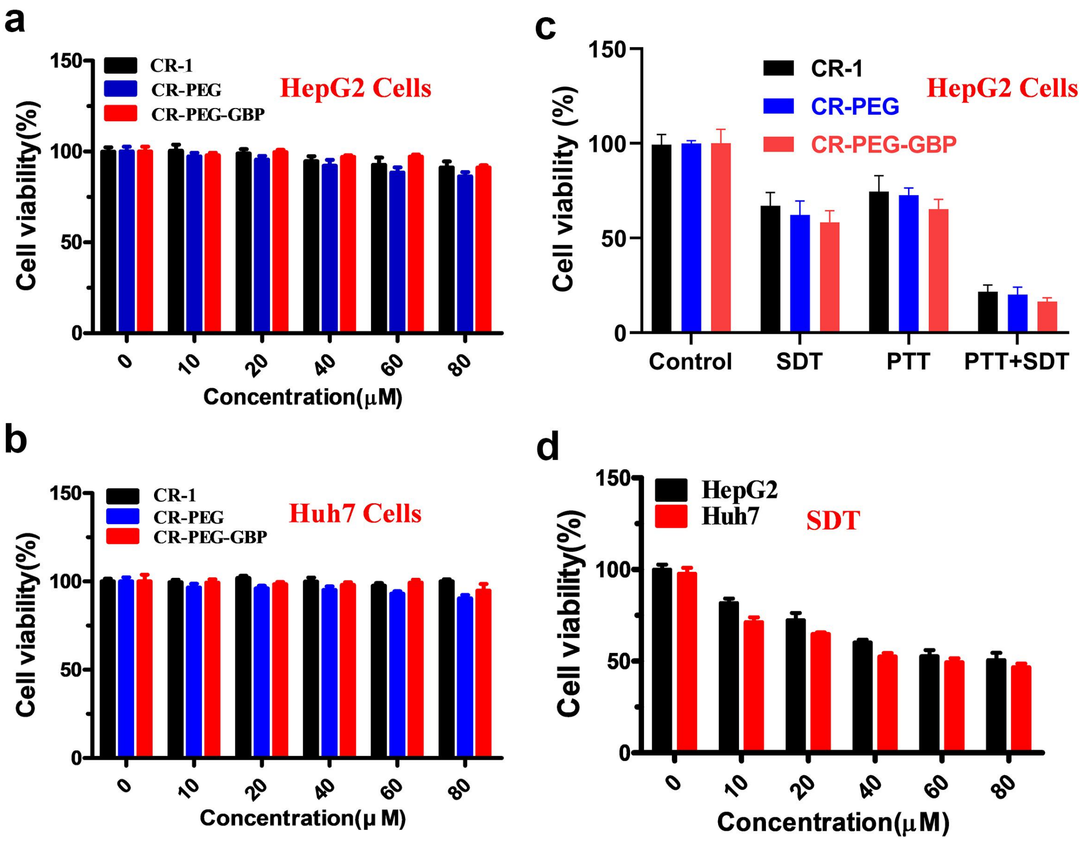

Figure S16. (a) and (b) *In vitro* viabilities of HepG2 cells (a) and Huh7 cells (b) incubated with different samples in varied concentrations (0, 10, 20, 40, 60, and 80 μM). (c) Killing ratio of different compounds (40 μM) to HepG2 cell with different treatments. (d) SDT effects on HepG2 cells and Huh7 cells treated with CR-PEG-GBP in various concentrations. (PTT: 808 nm laser, 0.5 W/cm^2, 5 min; SDT: 1 MHz, 1.61 W/cm^2, 5 min). Data are mean ± s.d. Two-sided unpaired t-test.

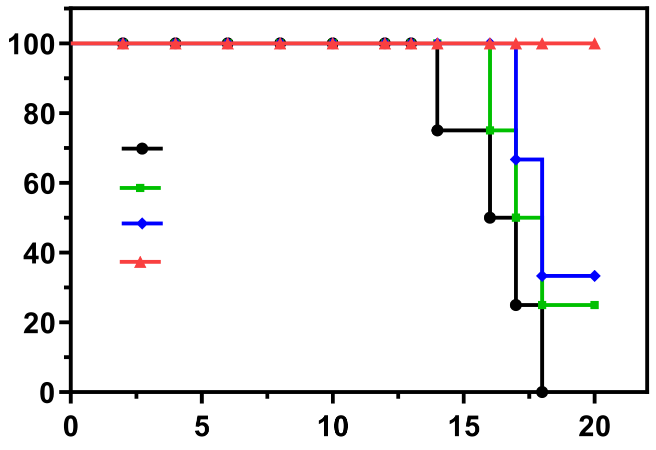

Figure S17. The survival rate of mice after different treatments.

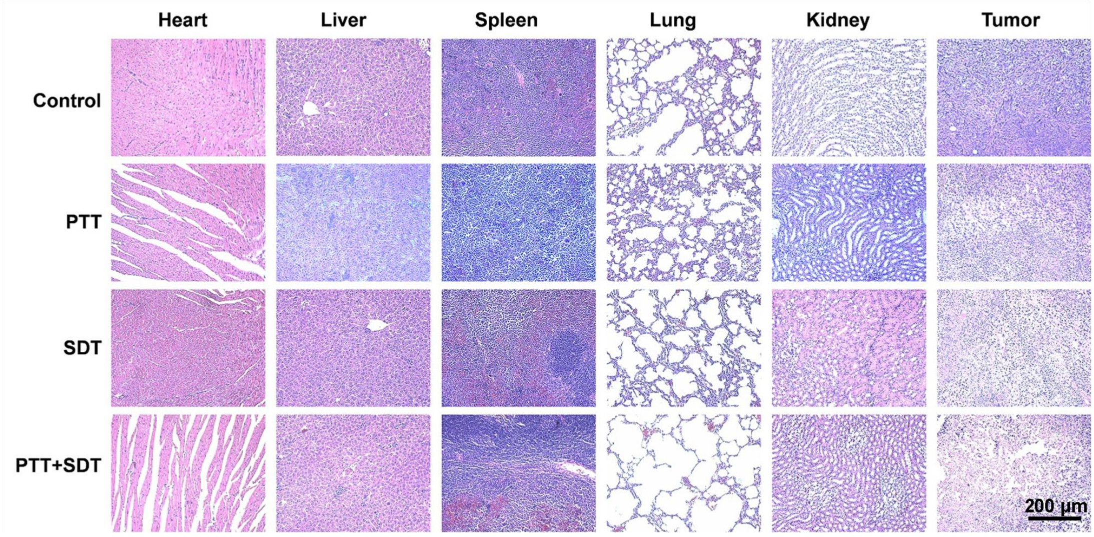

Figure S18. Biosafety evaluation of CR-PEG-GBP by H&E staining for tumor sections and other normal organs.
